# Efficacy and safety of 5 mg olanzapine for nausea and vomiting management in cancer patients receiving carboplatin: integrated study of three prospective multicenter phase II trials

**DOI:** 10.1186/s12885-021-08572-3

**Published:** 2021-07-19

**Authors:** Senri Yamamoto, Hirotoshi Iihara, Ryuji Uozumi, Hitoshi Kawazoe, Kazuki Tanaka, Yukiyoshi Fujita, Masakazu Abe, Hisao Imai, Masato Karayama, Yoh Hayasaki, Chiemi Hirose, Takafumi Suda, Kazuto Nakamura, Akio Suzuki, Yasushi Ohno, Ken-ichirou Morishige, Naoki Inui

**Affiliations:** 1 Department of Pharmacy, Gifu University Hospital, 1-1 Yanagido, Gifu, Gifu 501-1194 Japan; 2 Laboratory of Pharmacy Practice and Social Science, Gifu Pharmaceutical University, 1-25-4 Daigakunishi, Gifu, Gifu 501-1196 Japan; 3 Department of Biomedical Statistics and Bioinformatics, Kyoto University Graduate School of Medicine, 54 Kawahara-cho, Shogoin, Sakyo-ku, Kyoto, 606-8507 Japan; 4 Division of Pharmaceutical Care Sciences, Center for Social Pharmacy and Pharmaceutical Care Sciences, Keio University Faculty of Pharmacy, 1-5-30 Shibakoen, Minato-ku, Tokyo, 105-8512 Japan; 5 Division of Pharmaceutical Care Sciences, Keio University Graduate School of Pharmaceutical Sciences, 1-5-30 Shibakoen, Minato-ku, Tokyo, 105-8512 Japan; 6 Second Division, Department of Internal Medicine, Hamamatsu University School of Medicine, 1-20-1, Handayama, Hamamatsu, 431-3192 Japan; 7 Division of Pharmacy, Gunma Prefectural Cancer Center, 617-1 Takahayashi-nishi, Ota, Gunma 373-8550 Japan; 8 Division of Gynecology, Shizuoka Cancer Center, 1007 Shimonagakubo, Nagaizumi-cho, Sunto-gun, Shizuoka, 411-8777 Japan; 9 Present address: Department of Obstetrics and Gynecology, Hamamatsu University School of Medicine, 1-20-1, Handayama, Hamamatsu, 431-3192 Japan; 10 Division of Respiratory Medicine, Gunma Prefectural Cancer Center, 617-1, Takahayashi-nishi, Ota, Gunma 373-8550 Japan; 11 Present address: Department of Respiratory Medicine, Comprehensive Cancer Center, International Medical Center, Saitama Medical University, 1397-1, Yamane, Hidaka, Saitama, 350-1298 Japan; 12 Department of Clinical Oncology, Hamamatsu University School of Medicine, 1-20-1 Handayama, Hamamatsu, 431-3192 Japan; 13 Department of Obstetrics and Gynecology, Gifu University Graduate School of Medicine, 1-1 Yanagido, Gifu, Gifu 501-1194 Japan; 14 Department of Gynecology, Gunma Prefectural Cancer Center, 617-1, Takahayashi-nishi, Ota, Gunma 373-8550 Japan; 15 Department of Cardiology and Respiratory Medicine, Gifu University Graduate School of Medicine, 1-1 Yanagido, Gifu, Gifu 501-1194 Japan; 16 Department of Clinical Pharmacology and Therapeutics, Hamamatsu University School of Medicine, 1-20-1, Handayama, Hamamatsu, 431-3192 Japan

**Keywords:** Antiemetics, Carboplatin, Nausea, Olanzapine, Vomiting

## Abstract

**Background:**

The efficacy of olanzapine as an antiemetic agent in cancer chemotherapy has been demonstrated. However, few high-quality reports are available on the evaluation of olanzapine’s efficacy and safety at a low dose of 5 mg among patients treated with carboplatin regimens. Therefore, in this study, we investigated the efficacy and safety of 5 mg olanzapine for managing nausea and vomiting in cancer patients receiving carboplatin regimens and identified patient-related risk factors for carboplatin regimen-induced nausea and vomiting treated with 5 mg olanzapine.

**Methods:**

Data were pooled for 140 patients from three multicenter, prospective, single-arm, open-label phase II studies evaluating the efficacy and safety of olanzapine for managing nausea and vomiting induced by carboplatin-based chemotherapy. Multivariable logistic regression analyses were performed to determine the patient-related risk factors.

**Results:**

Regarding the endpoints of carboplatin regimen-induced nausea and vomiting control, the complete response, complete control, and total control rates during the overall study period were 87.9, 86.4, and 72.9%, respectively. No treatment-related adverse events of grade 3 or higher were observed. The multivariable logistic regression models revealed that only younger age was significantly associated with an increased risk of non-total control. Surprisingly, there was no significant difference in CINV control between the patients treated with or without neurokinin-1 receptor antagonist.

**Conclusions:**

The findings suggest that antiemetic regimens containing low-dose (5 mg) olanzapine could be effective and safe for patients receiving carboplatin-based chemotherapy.

## Background

Chemotherapy-induced nausea and vomiting (CINV) is the most distressing side effect of cancer chemotherapy [1, 2]. Furthermore, it can have a strong negative impact on patients’ quality of life (QOL) [3]. Carboplatin (CBDCA), a second-generation platinum compound, is a key drug for the treatment of a variety of cancers and used commonly. CBDCA with a target area under the curve [AUC] ≥ 4 mg/mL/min is classified as a moderate-emetic-risk chemotherapy (MEC) or high-emetic-risk chemotherapy (HEC) [4–7]. The latest international antiemetic guidelines recommend a three-drug combination comprising 5-hydroxytryptamine-3 receptor antagonist (5-HT_3_RA), dexamethasone (DEX), and neurokinin-1 receptor antagonist (NK_1_RA) as standard antiemetic prophylaxis for CINV in patients receiving CBDCA-based chemotherapy [4–7]. However, control of CBDCA-induced nausea and vomiting remains poor even with a standard triplet therapy and is associated with some patient-related risk factors such as female sex, younger age, and alcohol use (frequency or unit of alcohol per week) [8–11]. In patients with lung cancer, the complete response (CR) rate, defined as the absence of emetic episodes and no administration of rescue medication for CINV, has been reported to be 80–90% [12–15]. In contrast, in female patients or patients with gynecologic cancer receiving CBDCA, the CR rate was approximately 62% [16, 17]. Given this context, further efforts are warranted to control CBDCA-induced nausea and vomiting in female and younger patients to improve their QOL.

Olanzapine is an antipsychotic drug classified as a multi-acting, receptor-targeted agent that has various affinities for multiple receptors, including dopaminergic D_1_, D_2_, D_3_, and D_4_ receptors, serotonergic 5-TH_2_A, 5-HT_2_B, 5-HT_3_, and 5-HT_6_ receptors, histamine H_1_ receptors, and muscarinic acetylcholine M_1_, M_2_, M_3_, and M_4_ receptors [18]. It has been reported to be a highly effective antiemetic drug in patients receiving MEC and/or HEC [19–24]. However, thus far, most of the reports have been about the efficacy of olanzapine 10 mg in HEC. Navari et al. demonstrated that a four-drug combination including 10 mg olanzapine was superior to standard antiemetic triplet therapy for patients, but patients who received olanzapine had significantly more severe sedation than those receiving placebo [23]. To solve this problem, a comparative study of 5 and 10 mg of olanzapine was conducted, and 5 mg olanzapine was found to have a comparable effect with a lesser sedative effect [25]. The research group continued to conduct the J-FORCE study, which examined a four-drug combination including 5 mg olanzapine for patients receiving high-dose cisplatin [24]. This study demonstrated that 5 mg olanzapine was superior to the placebo and did not have a significant effect on daytime somnolence.

However, no large-scale trials have evaluated the efficacy of olanzapine in MEC, especially at a dose of 5 mg. The efficacy and safety of 5 mg olanzapine for antiemetic prophylaxis in patients receiving CBDCA-based chemotherapy has been reported in only three phase II studies [26–28]. Tanaka et al. and Iihara et al., respectively, reported the efficacy of a four-drug combination consisting of olanzapine, NK_1_RA, 5-HT_3_RA, and DEX in 33 patients with lung cancer and 57 patients with gynecological cancer [26, 27]. Sakai et al. reported the efficacy of a three-drug combination consisting of olanzapine, 5-HT_3_RA, and DEX in 50 patients with thoracic malignancies [28]. The overall CR rates in these trials were 78.9% [27], 93.9% [26], and 94.0% [28]. It is questionable whether the CR rate is comparable in patients having thoracic malignancies treated with or without NK_1_RA treatment and if it differs greatly between patients with lung cancer and those with gynecological cancer. Thus, this study was aimed at evaluating the efficacy and safety of 5 mg olanzapine for the management of nausea and vomiting in cancer patients receiving CBDCA-based chemotherapy based on data from three prospective multicenter phase II trials. Another objective of the study was to identify patient-related risk factors for CBDCA-induced nausea and vomiting treated with 5 mg olanzapine.

## Methods

### Study design

**Fig. 1:**
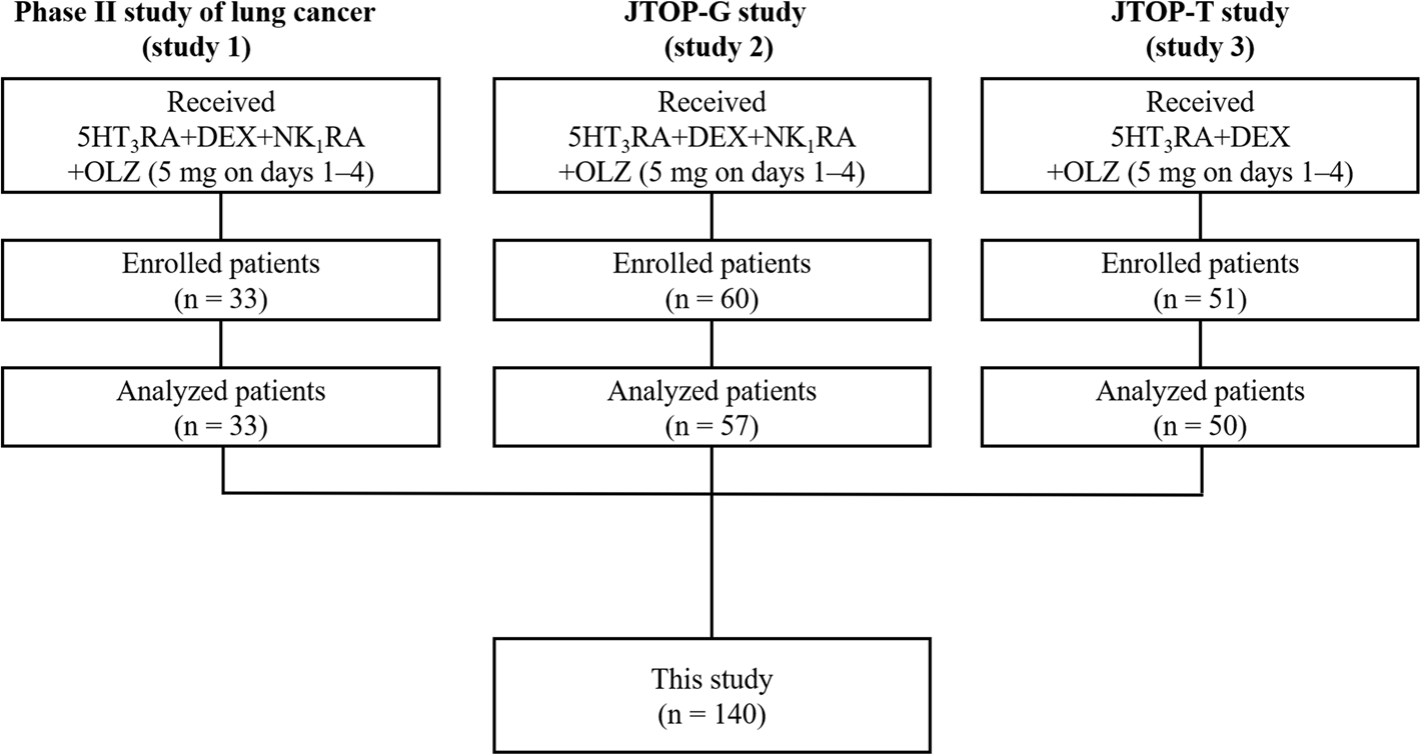
The study flow diagram. In all, 140 patients who received carboplatin-based chemotherapy were analyzed from three multicenter, prospective, single-arm, open-label, phase II studies. 5-HT_3_RA, 5-hydroxytryptamine-3 receptor antagonist; DEX, dexamethasone; NK_1_RA, neurokinin-1 receptor antagonist; OLZ, olanzapine

Pooled data of 140 patients from three multicenter, prospective, single-arm, open-label, phase II studies were analyzed. The results of these studies have been previously published [26–28]. A flow diagram of the present study is shown in Fig. 1. The studies in question were conducted in patients who were scheduled to receive CBDCA-based chemotherapy (AUC: ≥ 4 mg/mL/min). The antiemetic regimens used in the three studies are shown in Table 1. The studies were registered with the University Hospital Medical Information Network, number UMIN000026739 (Study 1), UMIN000031646 (Study 2), and UMIN000031267 (Study 3).

**Table 1:** Antiemetic regimens in each study

Antiemetic		Day 1	Day 2	Day 3	Day 4
**Study 1**					
5HT_3_RA	i.v.	※			
Aprepitant	p.o.	125 mg	80 mg	80 mg	
Dexamethasone*	i.v.	4.95 mg			
Olanzapine	p.o.	5 mg	5 mg	5 mg	5 mg
**Study 2**					
Granisetron	i.v.	1 mg			
Aprepitant	p.o.	125 mg	80 mg	80 mg	
Dexamethasone** or Dexamethasone	p.o.	12 mg			
	i.v.	9.9 mg			
Olanzapine	p.o.	5 mg	5 mg	5 mg	5 mg
**Study 3**					
Granisetron	i.v.	1 mg			
Dexamethasone ** or Dexamethasone	p.o.	12 mg	8 mg	8 mg	
	i.v.	9.9 mg	6.6 mg	6.6 mg	
Olanzapine	p.o.	5 mg	5 mg	5 mg	5 mg

Dexamethasone 3.3 mg i.v. = 4 mg p.o.

※Granisetron: 1 or 3 mg, palonosetron: 0.75 mg, ramosetron: 0.3 mg.

*When paclitaxel is used, 9.9 or 12 mg DEX is, respectively, administered intravenously or orally on day 1

**When more than 135 mg/m^2^ paclitaxel is used, 19.8 or 20 mg DEX is, respectively, administered intravenously or orally on day 1

*5-HT*_*3*_*RA* 5-hydroxytryptamine-3 receptor antagonist.

### Data collection

The patients enrolled in these three studies were aged ≥20 years; had lung cancer (Study 1), gynecologic cancer (Study 2), and thoracic malignancies (Study 3); and were chemotherapy-naïve (Study 1); and had no history of treatment with MEC and/or HEC (Studies 2 and 3). Data were collected from patients’ self-reported diaries. Patients completed a daily diary on days 1–5 (Studies 1 and 3) and days 1–7 (Study 2) from the initiation of CBDCA treatment. Patients reported experiencing nausea, somnolence, and decreased concentration by using a four-point scale (none, mild, moderate, and severe). Adverse events were graded according to the Common Terminology Criteria for Adverse Events (CTCAE) version 4.0. The data sources included patient-related risk factors such as sex, age, habitual alcohol consumption (defined as exceeding occasional drinking including occasionally consuming alcohol with meals or during social occasions, travel, parties etc.), motion sickness, morning sickness, and Eastern Cooperative Oncology Group performance status (ECOG PS). The endpoints were CR rate, which was defined as no emetic episodes and no administration of rescue medication for CINV; and complete control (CC) rate, which was defined as no emetic episodes, no use of rescue medication, and no significant nausea (defined as no more than mild nausea); and total control (TC) rate, which was defined as no emetic episodes, no use of rescue medication, and no nausea. The assessment periods for CINV were 0–120 h after CBDCA initiation (overall), 0–24 h after CBDCA initiation (acute), and 24–120 h after CBDCA initiation (delayed).

### Statistical analysis

Patient characteristics, rate of CINV control, and treatment-related adverse events were summarized using descriptive statistics or reported in terms of frequencies and proportions of total patients. Univariable and multivariable logistic regression analyses were performed to determine the patient-related risk factors associated with non-CR, non-CC, and non-TC during the overall study period. The cut-off age was determined using the Youden index in receiver operating characteristic (ROC) curve analysis [29]. Youden’s index was calculated as the maximum value using the following formula: sensitivity – (1 – specificity). Sensitivity analysis using three propensity score (PS) methods was also performed to reduce the effects of confounding factors. The PS of the co-administration of NK_1_RA (i.e., NK_1_RA inclusive regimen) was estimated for each patient using a logistic regression model which included age and sex [30]. In the PS-matching analysis, 1:1 matching without replacement (greedy nearest neighbor matching algorithm) with a caliper width equal to 0.2 of the standard deviation of the logit of the PS was applied to create a matched sample [31]. We also used PS-adjusted (including the PS as an additional covariate), and inverse probability of treatment weighting (IPTW) methods [32]. The results are shown as odds ratios (ORs) and 95% confidence intervals (CIs). All statistical analyses were performed using JMP 15.0.0 and SAS version 9.4 (SAS Institute, Inc., Cary, NC, USA). All *p*-values were two-sided, and statistical significance was set at a *P* value < 0.05.

## Results

### Study patients

Demographic data and patient characteristics are shown in Table 2. The median patient age was 68 years (range, 34–85 years). The numbers of female and male patients were 76 (54.3%) and 64 (45.7%), respectively. The number of patients who received NK_1_RA was 90 (64.3%).

**Table 2:** Patients’ characteristics

Total Number	140^a^	
Age (years)		
Median (range)	68 (34–85)	
	No.	%
Sex		
Female	76	54.3
Male	64	45.7
ECOG PS		
0	108	77.1
1	24	17.1
2	8	5.7
Cancer type		
Non-small-cell lung cancer	57	40.7
Ovarian cancer	28^b^	20.0
Endometrial cancer	22^b^	14.7
Small-cell lung cancer	20	14.3
Cervical cancer	7	5.0
Thymoma/thymic carcinoma	3	2.1
Others	4	2.9
Carboplatin dose		
AUC 6 mg/mL/min	73	52.1
AUC 5 mg/mL/min	66	47.1
AUC 4 mg/mL/min	1	0.7
Additional anticancer drugs		
Paclitaxel	58	41.4
Paclitaxel+Pembrolizumab	2	1.4
Paclitaxel+Bevacizumab+Atezolizumab	2	1.4
Nab-Paclitaxel	2	1.4
Nab-Paclitaxel+Pembrolizumab	4	2.9
Pemetrexed	10	7.1
Pemetrexed+Pembrolizumab	4	2.9
Pemetrexed+Bevacizumab	3	2.1
Etoposide	10	7.1
Etoposide+Atezolizumab	2	1.4
Vinorelbine	5	3.6
S-1	3	2.1
Docetaxel	2	1.4
NK_1_RA inclusive regimen		
Yes	90	64.3
No	50	35.7
Habitual alcohol consumption		
Yes	50	35.7
No	90	64.3
Motion sickness		
Yes	57	40.7
No	83	59.3
Morning sickness		
Yes	45	32.1
No	55	39.3
Unknown	40	28.6

^a^ Percentages may not add up to 100 because of rounding off

^b^ One patient had comorbidities of ovarian and endometrial cancer

*ECOG PS* Eastern Cooperative Oncology Group performance status; *AUC* area under the curve; *S-1* tegafur plus gimeracil plus oteracil potassium.

### Control of CINV

**Fig. 2:**
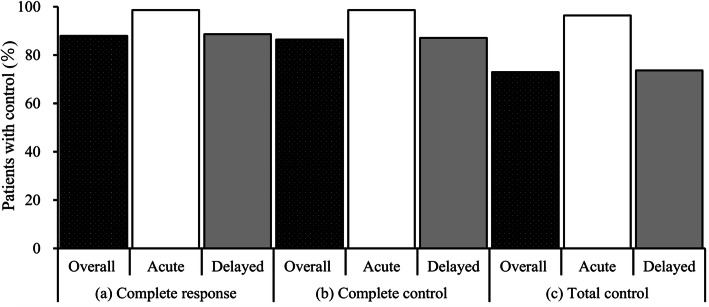
(**a**) Complete response, (**b**) complete control, and (**c**) total control. The bar graph shows the percentages of patients in whom chemotherapy-induced nausea and vomiting was controlled in the overall (0–120 h), acute (0–24 h), and delayed (24–120 h) periods

As shown in Fig. 2, the CR, CC, and TC rates during the overall period were 87.9, 86.4, and 72.9%, respectively. The corresponding rates during the acute period were 98.6, 98.6, and 96.4%. The CR, CC, and TC rates in the delayed period were 88.6, 87.1, and 73.6%, respectively. The rates of nausea, significant nausea, and vomiting during the overall period were 26.4, 4.3, and 8.6%, respectively. The corresponding rates during the acute period were 2.9, 0.7, and 0.7%. The rates of nausea, significant nausea, and vomiting during the delayed period were 25.7, 4.3, and 8.6%, respectively.

### Risk factors affecting CINV control

Using the ROC curve method, the cut-off values of age were predicted to be 55, 61, and 59 years for non-CR, non-CC, and non-TC, respectively, during the overall study period. The AUC values based on the ROC curve for non-CR, non-CC, and non-TC were 0.654, 0.629, and 0.677, respectively. For the present study, the cut-off value for age was set to 60 years. The risk analysis results for non-CR, non-CC, and non-TC during the overall study period are shown in Table 3. Multivariable logistic regression models showed that younger age was significantly associated with an increased risk of non-TC in the overall study period (adjusted OR, 3.08; 95% CI, 1.17–8.12; *P* = 0.023). In contrast, the co-administration of NK_1_RA, i.e., number of antiemetics, was not significantly associated with non-CR (adjusted OR, 1.43; 95% CI, 0.33–6.14; *P* = 0.628), non-CC (adjusted OR, 1.89; 95% CI, 0.46–7.78; *P* = 0.378), and non-TC (adjusted OR, 1.47; 95% CI, 0.49–4.46; *P* = 0.492) during the overall study period.

**Table 3:** Risk analysis for non-CR, non-CC, and non-TC during the overall study period

	Univariable analysis			Multivariable analysis		
	OR	95% CI	*P* value	OR	95% CI	*P* value
**A: non-CR**						
Sex (female vs male)	4.59	1.26–16.78	0.021	2.50	0.54–11.63	0.241
Age (< 60 years vs > =60 years)	4.24	1.49–12.09	0.007	2.40	0.69–8.37	0.169
NK_1_RA inclusive regimen (yes vs no)	2.89	0.79–10.58	0.110	1.43	0.33–6.14	0.628
CBDCA dose (6 vs 4–5)	2.44	0.81–7.34	0.113			
Habitual alcohol consumption (yes vs no)	0.35	0.09–1.27	0.110			
Motion sickness (yes vs no)	1.34	0.48–3.72	0.571			
Morning sickness (yes vs no)	2.16	0.65–7.15	0.206			
**B: non-CC**						
Sex (female vs male)	3.69	1.16–11.76	0.027	1.78	0.43–7.37	0.430
Age (< 60 years vs > =60 years)	4.17	1.53–11.34	0.005	2.54	0.75–8.55	0.134
NK_1_RA inclusive regimen (yes vs no)	3.39	0.94–12.25	0.063	1.89	0.46–7.78	0.378
CBDCA dose (6 vs 4–5)	2.20	0.79–6.18	0.133			
Habitual alcohol consumption (yes vs no)	0.30	0.08–1.07	0.063			
Motion sickness (yes vs no)	1.07	0.40–2.85	0.894			
Morning sickness (yes vs no)	1.48	0.49–4.46	0.483			
**C: non-TC**						
Sex (female vs male)	3.77	1.62–8.76	0.002	1.69	0.60–4.76	0.323
Age (< 60 years vs > =60 years)	5.06	2.26–11.33	< 0.001	3.08	1.17–8.12	0.023
NK_1_RA inclusive regimen (yes vs no)	3.23	1.30–8.01	0.012	1.47	0.49–4.46	0.492
ECOG PS (0–1 vs 2)	2.73	0.32–22.93	0.356			
CBDCA dose (6 vs 4–5)	2.54	1.15–5.57	0.021	1.46	0.57–3.72	0.431
Habitual alcohol consumption (yes vs no)	0.66	0.29–1.47	0.310			
Motion sickness (yes vs no)	0.69	0.32–1.49	0.340			
Morning sickness (yes vs no)	1.62	0.69–3.77	0.264			

*OR* odds ratio; *CI* confidence interval; *CR* complete response; *CC* complete control; *TC* total control; *CBDCA* carboplatin; *ECOG PS* Eastern Cooperative Oncology Group performance status.

### Sensitivity analysis of the co-administration of NK_1_RA

The risk analysis results for non-CR, non-CC, and non-TC during the overall study period are shown in Table 4. Except for IPTW analysis of non-TC, the other sensitivity analyses using the three PS methods showed similar results. The co-administration of NK_1_RA was not significantly associated with non-CR, non-CC, and non-TC during the overall study period.

**Table 4:** Sensitivity analysis of NK_1_RA co-administration for non-CR, non-CC, and non-TC during the overall study period

	PS-matched analysis			PS-adjusted analysis			IPTW		
	OR	95% CI	*P* value	OR	95% CI	*P* value	OR	95% CI	*P* value
**A: non-CR**									
NK_1_RA inclusive regimen (yes vs no)	2.10	0.36–12.11	0.406	1.50	0.36–6.31	0.579	2.25	0.59–8.58	0.233
**B: non-CC**									
NK_1_RA inclusive regimen (yes vs no)	2.69	0.49–14.69	0.253	1.95	0.48–7.89	0.348	2.74	0.70–10.67	0.146
**C: non-TC**									
NK_1_RA inclusive regimen (yes vs no)	1.41	0.44–4.46	0.561	1.77	0.61–5.11	0.293	2.68	1.07–6.72	0.036

*OR* odds ratio; *CI* confidence interval; *CR* complete response; *CC* complete control; *TC* total control; *PS* propensity score; *IPTW* inverse probability of treatment weighting.

### Safety

**Fig. 3:**
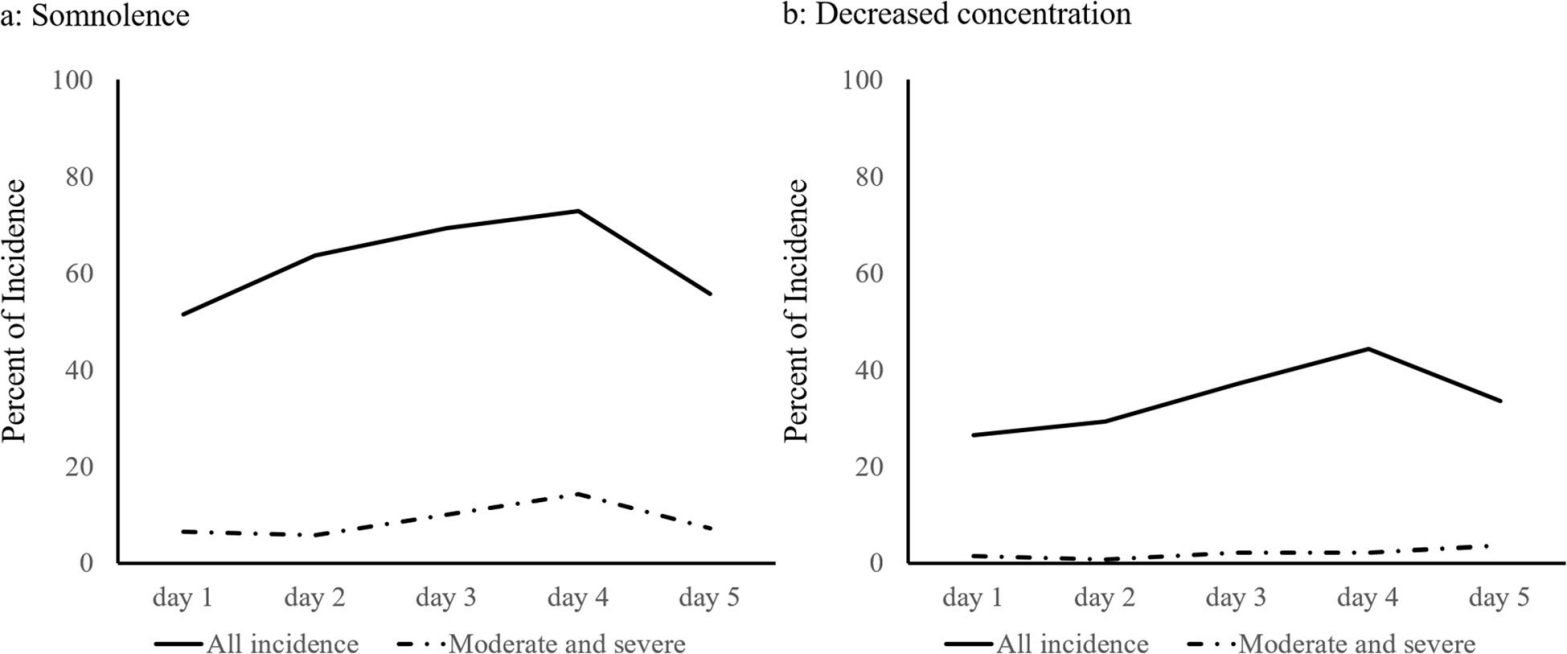
Incidences of somnolence and decreased concentration for 5 days after the initiation of chemotherapy

The treatment-related adverse events associated with olanzapine administration are shown in Table 5. Evaluation based on CTCAE version 4.0 revealed that the rates of grade 2 somnolence were as low as 2.9%, and there were no instances of somnolence of grade 3 or higher. The assessment of patients’ self-reported diaries, wherein they rated their symptoms using a four-point scale (none, mild, moderate, and severe), revealed the incidence of somnolence and decreased concentration were 85.7 and 60.0%, respectively (Fig. 3). The incidence of moderate and severe somnolence was 22.1%, while that of decreased concentration was only 7.1%.

**Table 5:** Treatment-related adverse events

Symptom	CTCAE v4.0					
	Any grade		Grade 1		Grade 2	
	No.	%	No.	%	No.	%
Dry mouth	69	49.3	66	47.1	3	2.1
Hiccups	47	33.6	42	30.0	5	3.6
Constipation	97	69.3	69	49.3	28	20.0
Dizziness	41	29.3	41	29.3	0	0.0
Insomnia	62	44.3	58	41.4	4	2.9
Somnolence	96	68.6	92	65.7	4	2.9

*CTCAE v4.0* Common Terminology Criteria for Adverse Events version 4.0.

## Discussion

In this integrated analysis, antiemetic therapy with 5 mg olanzapine showed a high CR rate against CBDCA-induced nausea and vomiting. Moreover, the treatment had an acceptable safety profile. Navari et al. reported a head-to-head comparison of the effect of 10 mg olanzapine versus aprepitant (fosaprepitant) when combined with palonosetron and DEX for cyclophosphamide/doxorubicin or cisplatin-based regimens in two phase III trials [19, 33]. In these two studies, there were no significant differences in CR between the olanzapine and aprepitant (fosaprepitant) regimens in any evaluation period. In contrast, treatment with 10 mg olanzapine resulted in significantly higher control of nausea in the delayed and overall periods than that of aprepitant (fosaprepitant). In this integrated analysis, nausea was observed in only 26.4% of the patients, with a particularly significant nausea (moderate-to-severe nausea) rate of only 4.3%, indicating excellent nausea control. The findings of this study are in line with those of previous studies on 10 mg olanzapine.

In the present study, we analyzed the risk factors affecting CINV control using non-CR, non-CC, and non-TC during the overall study period. In the multivariable analyses, the only patient-related risk factors detected even with the addition of olanzapine were younger age for patients with non-TC. Younger age is a well-known risk factor for CINV [8–11]. This suggests that the combination of olanzapine may be able to counteract patient-related risk factors in cases of severe CINV. In the present study, the cut-off value for age was set to 60 years for non-TC in the overall period based on the ROC curve method. This is consistent with our previous analysis of the age cut-off for nausea in 608 patients who received the first cycle of chemotherapy [34]. Therefore, further development of antiemetic therapy is needed to completely control nausea, especially in younger patients.

To the best of our knowledge, there are no studies that have evaluated the efficacy of adding NK_1_RA to antiemetic therapy consisting of olanzapine, 5HT_3_RA, and DEX in MEC and HEC. Multivariable analysis showed that there was no statistically significant difference in CINV control between the patients treated with or without NK_1_RA. Sensitivity analysis using PS-matched, PS-adjusted, and IPTW methods yielded consistent results, except for the IPTW result in non-TC. Therefore, except in young patients, olanzapine may be used in combination with NK_1_RA de-escalation, which appears to be a reasonable treatment approach as a prophylactic antiemetic for CBDCA-based chemotherapy. Thus, this should be confirmed with a randomized comparison including older and younger patients in future research.

The question remains as to why NK_1_RA might be omitted by combining olanzapine here. Recently, the efficacy of mirtazapine, an antidepressant that has affinity for serotonin (5-TH_2A_, 5-HT_2C_, 5-HT_3_, 5-HT_6_), histamine (H_1_), adrenaline (ɑ_1_), and muscarinic receptors, as well as olanzapine, has been reported as an antiemetic treatment for cancer chemotherapy [35, 36]. Stimulation of 5HT_2_ or 5HT_1A_ by mirtazapine and the interactions between mirtazapine and neurokinin-1 have also been shown. Furthermore, the drug may exert its anti-nausea and antiemetic effects indirectly by inhibiting the NK-1 receptor [37]. However, the effect of olanzapine on the NK_1_ receptor is not clear and warrants further investigation.

Excessive sedation is an adverse event that should be noted when administering 10 mg olanzapine. No grade 3 or higher somnolence was observed in this integrated analysis. Patient diary reports of somnolence ranged from 51.4 to 72.9% over 5 days. The percentage of patients with moderate-to-severe somnolence ranged from 5.7 to 14.3%. In the J-FORCE study, which examined a four-drug combination including 5 mg olanzapine for patients receiving high-dose cisplatin, the overall incidence of somnolence in the olanzapine group ranged from 70.5 to 76.6%, with the incidence in the moderate-to-severe group being 11.0 to 14.2% [24]. The incidence of somnolence in the placebo group in the J-FORCE study ranged from 67.8 to 76.7% overall and from 7.4 to 19.5% in the moderate-to-severe group. Our results are comparable to those of the J-FORCE study and suggest that olanzapine therapy administered 4 days after dinner does not have a significant effect on daytime somnolence. With regard to decreased concentration due to somnolence, the overall rate was 26.4 to 44.3%, while that for moderate-to-severe somnolence was 0.7 to 3.6%. In the J-FORCE study, the incidence of decreased concentration was comparable between the olanzapine group (overall: 40.4 to 51.0%, moderate to severe: 4.8 to 7.9%) and placebo group (overall: 35.6 to 55.7%, moderate to severe: 4.0 to 14.1%). This suggests that olanzapine administration had no effect on the difficulty experienced in daily life.

The present study has some limitations. First, all the studies included in this integrated analysis had an open-label and single-arm design. Second, three phase II studies used quite broad definition of the habitual alcohol consumption [26–28]. In CINV studies, alcohol use has traditionally been as weekly frequency of consumption [drinks/week]; no, rarely, occasionally, regularly, sometimes, every day or units/weeks [8–11]. The present study used the same definition as J-FORCE study, a large phase III trial of 5 mg olanzapine [24]. Furthermore, the results were obtained only in the Japanese population, and thus, they may not be extrapolatable to patients globally.

However, the present study showed that 5 mg olanzapine combined with 5-HT_3_RA and DEX with/without NK_1_RA could be an effective and safe standard treatment for patients treated with CBDCA-based chemotherapy with an AUC ≥ 4 mg/mL/min. In the future, a phase III trial comprising a head-to-head comparison of the efficacy and safety of 5 mg olanzapine versus NK_1_RA when combined with 5-HT_3_RA and DEX for patients receiving CBDCA-based chemotherapy is warranted.

## Conclusion

In conclusion, a low dose of 5 mg olanzapine combined with 5-HT_3_RA and DEX with/without NK_1_RA could be an effective and safe standard treatment for patients treated with an AUC of ≥4 mg/mL/min of CBDCA-based combination chemotherapy.

## Data Availability

The data that support the findings of this study are available from the study groups of study 1, study 2, and study 3 but restrictions apply to the availability of these data, which were used under license for the current study, and therefore, the data are not publicly available. However, data are available from the authors upon reasonable request and with permission of the study groups.

## Abbreviations

5-HT_3_RA: 5-Hydroxytryptamine-3 receptor antagonist
AUC: Area under the curve
CBDCA: Carboplatin
CC: Complete control
CI: Confidence interval
CINV: Chemotherapy-induced nausea and vomiting
CR: Complete response
CTCAE: Common terminology criteria for adverse events
DEX: Dexamethasone
ECOG PS: Eastern cooperative oncology group performance status
HEC: High-emetic-risk chemotherapy
IPTW: Inverse probability of treatment weighting
MEC: Moderate-emetic-risk chemotherapy
NK_1_RA: Neurokinin-1 receptor antagonist
OR: Odds ratio
PS: Propensity score
ROC: Receiver operating characteristic
S-1: Tegafur plus gimeracil plus oteracil potassium
TC: Total control
